# School experiences and self‐harm in the OxWell study

**DOI:** 10.1002/jcv2.70025

**Published:** 2025-07-02

**Authors:** Rasanat Fatima Nawaz, Tamsin Jane Ford, Mina Fazel, Galit Geulayov, The OxWell Study Team, Simon R. White

**Affiliations:** ^1^ Department of Psychiatry University of Cambridge Cambridge UK; ^2^ Department of Psychiatry University of Oxford Oxford UK; ^3^ MRC Biostatistics Unit University of Cambridge Cambridge UK

**Keywords:** mental health, schools, self‐harm, young people

## Abstract

**Background:**

Schools are key for identifying challenges faced by young people who self‐harm (SH). Understanding how school factors influence SH predictors is essential for developing effective school‐based interventions. We aimed to conduct a secondary data analysis using the OxWell Student Survey to identify associations between young people's school experiences and SH.

**Methods:**

Using cross‐sectional data from English secondary schools in the 2023 OxWell Student Survey, we conducted multi‐level logistic regressions to analyse whether SH was associated with student age, gender, mental health (RCADS) and wellbeing (sWEMWBS). School experience measures included enjoyment, bullying, racism, extracurricular activities, school worry, and adults listening.

**Results:**

Individual students' perception that the school did not deal well with bullying were associated with a 38% increase in SH (OR = 1.38; CI 1.20–1.59) and schools not dealing well with racism was associated with a 20% increase in the likelihood of SH (OR = 1.20; CI 1.04–1.38). Similarly, the likelihood of SH was 30% higher in schools with students feeling unfairly picked on by their teacher (OR = 1.30; CI 1.14–1.47). Greater SH was associated with being female (OR = 1.15; CI 0.99–1.32), gender diverse (OR = 3.49; CI 2.38–5.12), or preferring not to say (OR = 2.02; CI 1.44–2.83) compared to males. Lower wellbeing scores (OR = 0.93; CI 0.93–0.95) and higher RCADS scores (OR = 1.12; CI 1.11–1.13) were also linked to higher SH likelihood.

**Conclusion:**

Interventions that address bullying, racism, teacher‐pupil relationships as well as providing specific support for more vulnerable groups such as females and gender diverse young people are important components of public mental health interventions that might reduce levels of SH. Future research should explore these relationships longitudinally.

## Graphical abstract

Understanding which school experiences are associated with greater self‐harm. More support is needed for girls and gender diverse populations. Many school experiences are things we can address and change, such as bullying.

**Key points**

**What's known, what's new, and what's relevant?**

- Self‐harm (SH) is common among children and young people and is strongly associated with an increased risk of suicide.
- Schools are an ideal setting to identify and support young people who SH as well as for prevention.
- Specific perceptions linked to SH included feeling that school poorly manages bullying and racism, feeling picked on by a teacher, and feeling that adults in school do not listen to students' opinions.
- Higher anxiety and depression levels, and being female or gender diverse were associated with increased likelihood of SH.
- Findings are relevant to policy and service development by underscoring the need for targeted interventions addressing school climate, teacher‐student relationships, and support systems within educational settings.

## INTRODUCTION

Self‐harm (SH), including intentional self‐poisoning or self‐injury, is common among children and young people, and is often repeated, and strongly associated with suicide (Pisinger et al., 2019; Witt et al., 2020). Young people who engage in SH have a higher rates of suicide attempts and an increased likelihood of dying from suicide (Pisinger et al., 2019). Understanding risk and protective factors as well as mechanisms in SH is an important part of the process of identifying targets for the development of preventive interventions that may reduce the rates of SH among young people. Many young people who SH do not present to health service, underscoring the importance of community‐based studies in identifying associated risk factors, as well as schools as a setting for this work (Kidger et al., 2012; Nawaz et al., 2021).

The school environment plays a crucial role in shaping the health and well‐being of young people (Fazel et al., 2014; Ford et al., 2021). School is where young people spend a significant amount of their time, form friendships, and navigate social norms and academic expectations. With education offering long‐term and widespread accessibility, schools are a key setting for the implementation of effective interventions aimed at supporting well‐being, preventing mental health issues, and addressing identified challenges (Department of Health & Department of Education, 2017). Understanding whether school factors influence the likelihood that pupils SH, and if so what aspects of the school experience are involved, has important implications for the potential targets of school‐based interventions (Evans & Hurrell, 2016).

Although school staff are often very concerned about SH and keen to support early identification and timely support for those at risk of SH, they are reticent to explicitly discuss SH with the student population due to fears of contagion and concerns about maintaining the balance between raising awareness and promoting SH (Duncan et al., 2019; Evans et al., 2019). Studies of interventions in schools that aim to reduce SH are mostly aimed at teaching staff in secondary (high) schools (Pierret et al., 2020). Interventions include setting up in‐person and online workshops to increase teachers understanding of how to recognise and respond to SH, with mixed effectiveness (Glennon et al., 2020; Groschwitz et al., 2017; Townsend et al., 2018). Interventions directed at SH in schools are scarce (Nawaz et al., 2023) with school‐based interventions mainly focussing on suicide prevention for example, Saving and Empowering Young Lives in Europe (SEYLE; Wasserman et al., 2010) and signs of suicide (Aseltine & DeMartino, 2004; Schilling et al., 2014).

Several school‐related factors have already been identified as potential influences on the likelihood of SH in young people. Poor relationships with peers and teachers, poor attendance or disengagement from school, and reduced academic performance are all linked to an elevated risk of SH (Bowes et al., 2015; Epstein et al., 2020; Evans et al., 2019; Winsper et al., 2012). Additionally, disliking school at the age of 14 has been identified as a predictor of SH at age 16 (Kidger et al., 2015). Conversely, experiencing a sense of connection or engagement with school and feeling secure in the school environment are associated with a decreased risk of SH (Nawaz et al., 2023). These findings underscore the potential role that fostering positive school experiences might play in mitigating the risk of SH in young people among other adverse outcomes.

The aim of this study was to conduct a secondary analysis of data from the OxWell Student Survey to identify associations between young people's school experiences and SH. Specifically, our research aimed to answer the following questions:

1. To what extent does the level of reported SH vary across schools?
2. What school and individual level characteristics are associated with reports of SH in school?

## METHODS

The OxWell Student Survey is a repeated cross‐sectional survey of primary and secondary schools, and further education colleges (FECs) in England. The survey asks students a range of questions about mental health and wellbeing, life experiences, and behaviours, with age‐appropriate schedules for primary school (Years 5 and 6, ages 9–11 years) and secondary school/FECs (Years 7–13, ages 11–18 years). The OxWell Student Survey 2023 recruited 43,735 students, of whom 37,952 provided assent (for those under 16) or consent (for those aged 16 and over). The survey was conducted across 180 primary schools, secondary schools, and FECs. The SH question differed for students under the age of, therefore data from primary schools were excluded from the analyses. Additionally, data from the 10 FECs were excluded, as this study focuses specifically on school experience variables, which were not always applicable to students in further education. At the time that these data were collected in 2023, participants were enroled in school without changes in their learning resulting from COVID‐19 lockdowns or virtual learning.

Publicly available data on schools were gathered from the Department for Education (DfE; Gov.uk, 2020) and Office for National Statistics (2021). These data were linked to the OxWell study schools through a unique school identifier.

### Measures

#### Outcome measure

The SH outcome measure was developed by the OxWell study team and based on prior research into SH (Hawton et al., 2002). Self‐harm was measured using the following question:

‘Have you ever deliberately self‐harmed (e.g., by taking an overdose or injuring yourself on purpose in some way)?’

Response options included ‘yes’, ‘no’, ‘prefer not to say’ and ‘not sure what this means’. Participants who answered ‘prefer not to say’ and ‘not sure what this means’ were excluded from the current analysis as their SH status could not be confidently ascertained.

#### Individual measures

The OxWell data analyses included measures of SH, gender, age, mental wellbeing, anxiety and depression, and multiple measures relating to school experience.

Self‐harm was measured using the following question: *Have you ever deliberately self‐harmed (e.g., by taking an overdose or injuring yourself on purpose in some way)?* Response options included ‘yes’, ‘no’, ‘prefer not to say’ and ‘not sure what this means’. Participants who answered ‘prefer not to say’ and ‘not sure what this means’ were excluded from analyses as their reports of SH could not be definitively grouped into ‘yes’ or ‘no’, leaving it unclear whether SH occurred or not.

Students reported their demographic data such as year group and were asked ‘What is your gender?’ for which they could select, ‘female’, ‘male’, ‘other’, and ‘prefer not to say’. These groups were kept the same throughout all analyses as the sample size was sufficient in each group.

The short Warwick‐Edinburgh Mental Wellbeing Scale, WEMWBS (Clarke et al., 2011), was used to measure mental wellbeing. The scale consists of seven questions on a five‐point Likert scale, from which responses are added to generate a summary score between 7 and 35. Higher scores indicate greater mental wellbeing (Ringdal et al., 2018).

The 11–item Revised Children's Anxiety and Depression Scale (RCADS–11) was used to assess mental health difficulties (Radez et al., 2021). All statements have four response categories according to frequency (0—‘never’, 1—‘sometimes’, 2—‘often’, 3—‘always’). Responses are collated and turned into a Total Anxiety and Depression score (0–75), with higher scores indicating greater difficulties (Chorpita et al., 2022).

Two types of school data were available: publicly available administrative data about school characteristics and aggregated individual data to assess school experience. Publicly available data on schools were gathered from the Department for Education (DfE; Gov.uk, 2020) and Office for National Statistics (2021). These data included type of school, school sex, urbanicity, percentage of students within a school eligible for Free School Meals (FSM), Ofsted ratings, number of students in each school, and the Index of Multiple Deprivation (IMD), where lower scores show greater area‐level deprivation.

#### School measures

Publicly available data on schools were gathered from the Department for Education (DfE; Gov.uk, 2020) and Office for National Statistics (2021). These data included type of school, co‐education/single sex admission policies, urbanicity, percentage of students within a school eligible for FSM, Office for Standards in Education, Children's Services and Skills (OFSTED) ratings, number of students in each school, and the IMD taken from the school postcode, where lower scores show greater area‐level deprivation.

In addition, we dichotomised responses for nine school experience measures at the individual level, to estimate various aspects of student school experience and the wider school culture across the student body (see Table 1). Table 1 illustrates our conservative approach to the categories, which aimed to ensure that we did not overestimate the school experiences whether positive or negative. For example, if a student answered neither agree or disagree to ‘I enjoy school’, they were grouped into the disagree category as the answer was not a clear agree.

**TABLE 1:** School experience measures and the method of dichotomising variables.

Variable	Original response	Dichotomised response
I enjoy my school/college	Agree, strongly agree	Agree
	Strongly disagree, disagree, neither agree nor disagree	Disagree
How well does your school deal with bullying?	Average, quite well, extremely well	Well
	Very badly, not very well	Not well
My school/college has lots of activities (like sport and drama) to take part in at lunchtime or after school/college	Agree, strongly agree	Agree
	Strongly disagree, disagree, neither agree nor disagree	Disagree
Do you know who provides mental health support in your school/college (where to go when you are worried and want to talk to an adult)?	Yes	Yes
	No, not sure	No
I feel like I am part of my school	Agree, strongly agree	Agree
	Strongly disagree, disagree, neither agree nor disagree	Disagree
My school deals well with racism	Agree, strongly agree	Agree
	Strongly disagree, disagree, neither agree nor disagree	Disagree
I feel unfairly picked on by a teacher	Often, sometimes	Often
	Never/rarely	Rarely
Adults in my school listen when I share my opinion	Often, sometimes	Often
	Never/rarely	Rarely
I worry about going to school/college	Agree, strongly agree	Agree
	Strongly disagree, disagree, neither agree nor disagree	Disagree

### Data analysis

Analyses were performed in R version 4.3.1 (R Core Development Team, 2021). First, we describe the level of SH between schools and then we explored individual factors and whether individual school experiences accounted for variation in SH. Our analysis focussed on both school experience and instances of student SH, considering the hierarchical structure of students within schools as students from the same school may have more in common with each other than with students from other schools. We employed multilevel regression models with schools as a random effect to account for this nested structure. The multi‐level logistic regression model was conducted using the R *lme4* package (Bates et al., 2015) to examine the relationship between SH and the school experience variables. We included random intercepts for schools to account for potential clustering or correlation of responses within the same school. Only complete case observations with recordings for all the model parameters were considered to ensure model accuracy.

## RESULTS

**FIGURE 1:**
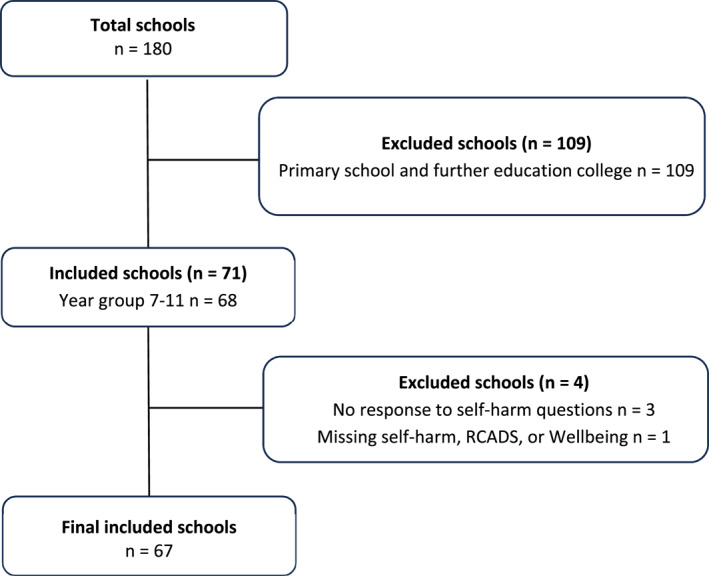
The inclusion and exclusion of schools in the analyses.

The final sample included 12,554 students in years 7–11 across 67 English secondary schools, as illustrated in Figure 1.

OxWell demographic data for the number of participants across gender, age, ethnicity, mental health and wellbeing is reported in Table 2. Gender was reported for 12,554 participants, with the breakdown as follows: Female (*N* = 6569), Male (*N* = 5310), Other (*N* = 262), and Prefer not to say (*N* = 413), with 0% missing data. Age ranged from 11 to 16 years, with the highest number of participants aged 13 (*N* = 2954), followed by 12 (*N* = 2921) and 14 (*N* = 2369). There were 73 participants with missing age data (0.58%). Ethnicity data were available for 12,554 participants, with the highest proportion identifying as White (*N* = 8231), followed by Asian/Asian British (*N* = 2225), Mixed/Multiple ethnic groups (*N* = 837), Black/Black British/African/Caribbean (*N* = 667), other ethnic group (*N* = 330), and Arab (*N* = 264). There were 0% missing ethnicity data. For mental health measures, Wellbeing (SWEMWS) data were available for 11,619 participants, with 935 missing responses (7.45%). RCADS (anxiety and depression measure) data were available for 11,849 participants, with 705 missing responses (5.62%).

**TABLE 2:** OxWell demographic data for the number of participants across gender, age, ethnicity, mental health and wellbeing (schools *N* = 67; participants *N* = 12,554).

Demographic variable	Number of participants
Gender	
Female	6569
Male	5310
Other	262
Prefer not to say	413
Missing	0
Age	
11	1309
12	2921
13	2954
14	2369
15	1887
16	1041
Missing	73
Ethnicity	
Arab	264
Asian/Asian British	2225
Black/Black British/African/Caribbean	667
Mixed/Multiple	837
White	8231
Other ethnic group	330
Missing	0
Mental health	
Wellbeing (SWEMWBS)	11,619
Missing	935
Anxiety and Depression Scale (RCADS)	11,849
Missing	705

School characteristics are presented in Table 3 for the 67 schools. The average number of students per school was 1092.75 (SD = 474.49). In terms of socio‐economic characteristics, the schools had a median IMD score of 6 (on a scale of 1–10), with an interquartile range of 7. The mean percentage of students eligible for FSM was 21.48% (SD = 16.88%).

**TABLE 3:** Characteristics of participating schools *n* = 67.

Characteristic		
Number of students mean (SD)		1092.75 (474.49)
Index of multiple deprivation median (IQR)		6 (7)
FSM % mean (SD)		21.48 (16.88)
Urbanicity (%)	Urban	93
	Rural	7
Type of school %	State secondary	68
	Independent schools	7
	Alternative provision	3
	Selective (faith and grammar)	22
School sex %	Mixed	86
	Female only	10
	Boys only	4
Ofsted %	Good	60
	Outstanding	18
	Requires improvement	9
	Inadequate	2
	Special measures	4

Regarding geographical location, a majority of the schools (93%) were located in urban areas, while only 7% were in rural settings. The sample primarily consisted of state secondary schools (68%), with smaller proportions of independent schools (7%), alternative provision schools (3%), and selective (faith and grammar) schools (22%). The majority of schools were mixed‐sex (86%), with 10% being girl‐only schools and 4% being boy‐only schools.

For school performance, 60% of schools were rated ‘Good’ by Ofsted, while 18% were rated ‘Outstanding’. A smaller proportion of schools were rated as ‘Requires improvement’ (9%), ‘Inadequate’ (2%), or were placed in ‘Special measures’ (4%).

Student data for gender and ethnicity were as follows. Females accounted for 53% of the sample, and 42% were males, 2% reported their gender as other, and 3% selected prefer not to say. The majority of students in the OxWell sample were White (65%), with 18% identifying as Asian/Asian British, 7% Mixed/Multiple Ethnic Groups, 5% Black/Black British/African/Caribbean, 2% Arab and 3% other ethnic groups.

Females accounted for 53% of the sample; 42% were males, 2% reported their gender as other, and 3% selected prefer not to say. Most students were White (65%), with 18% identifying as Asian/Asian British, 7% Mixed/Multiple Ethnic Groups, 5% Black/Black British/African/Caribbean, 2% Arab and 3% other ethnic groups.

### Variation of self‐harm across schools

**FIGURE 2:**
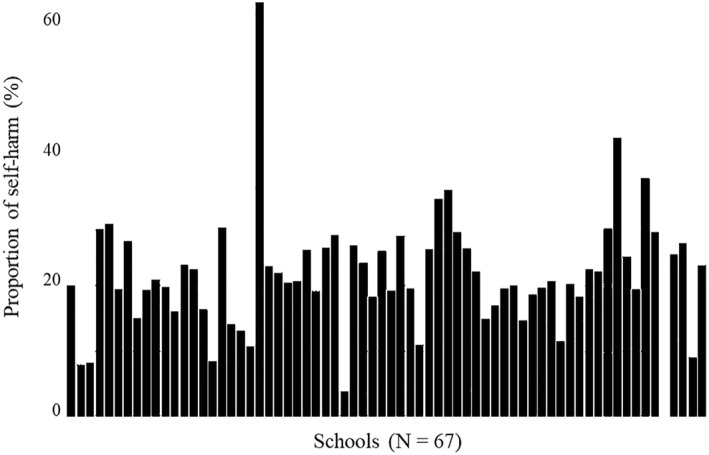
Proportion of self‐harm across schools (*N* = 67) in OxWell.

In the overall sample, 21% of students reported SH and 79% reported no SH. The proportion of pupils reporting SH within each school ranged from 0% to 63%, with a mean proportion of 22% (SD = 9%) and a median proportion of 21% (see Figure 2). These values highlight the variability in SH prevalence in the OxWell data across 67 different schools.

### Individual school experiences associated with self‐harm

In Table 4, the univariable logistic regression is adjusted for SH and a single variable of interest and uses the maximum available SH sample for each variable. The fully adjusted model controls for SH and adjusts for demographic variables (gender, school year), psychological variables (wellbeing and anxiety//depression scores) and accounts for clustering within schools (*N* = 67) through a random intercept for each school. The multivariable model is based on complete case data (*N* = 10,759). The fully adjusted model found individual students' perception that the school did not deal well with bullying was associated with a 38% increase in the likelihood of SH (adj. OR = 1.38; 95% CI 1.20–1.59, *p* < 0.001) and schools not dealing well with racism was associated with a 20% increase in the likelihood of SH (adj. OR = 1.20; 95% CI 1.04–1.38, *p* = 0.01). Similarly, schools with a higher proportion of students who often felt unfairly picked on by their teacher were associated with a 30% increase in the likelihood of SH (adj. OR = 1.30; 95% CI 1.14–1.47, *p* < 0.001). Additionally, schools with a higher proportion of students who reported that adults in school rarely listened to their opinion had a 21% increase in the likelihood of SH (adj. OR = 1.21; 95% CI 1.03–1.43, *p* = 0.02). Students who did not know who provides mental health support have a 31% lower likelihood of SH compared to students who do know (adj. OR = 0.69; 95% CI 0.61–0.79, *p* < 0.001).

**TABLE 4:** Logistic regression for school experience and self‐harm (SH), univariable model and fully adjusted model (*N* = 10,759).

Variable/correlate	SH ‘yes’	SH ‘no’	Univariable odds ratio [95% CI]	Fully adjusted odds ratio [95% CI]
Gender: What is your gender?				
Males	290	4026	Ref	Ref
Females	1796	4040	**2.59 [2.33–2.88]**	**1.15 [0.99–1.32]**
Other	184	73	**13.86 [10.49–18.31]**	**3.49 [2.38–5.12]**
Prefer not to say	195	155	**6.29 [ 4.95–7.98]**	**2.02 [1.44–2.83]**
School year 7–11	2355	8404	**1.15 [1.12–1.18]**	1.03 [1.04–1.11]
Wellbeing (S)WEMWBS	2355	8404	**0.82 [0.81–0.83]**	**0.93 [0.92–0.95]**
Anxiety and Depression (RCADS)	2355	8404	**1.17 [1.16–1.17]**	**1.12 [1.11–1.13]**
I enjoy my school/college				
Agree	580	4352	Ref	Ref
Disagree	1775	4052	**2.89 [2.62–3.18]**	1.11 [0.95–1.30]
How well does your school deal with bullying?				
Well	831	5272	Ref	Ref
Not well	1525	3131	**3.12 [2.84–3.43]**	**1.38 [1.20–1.59]**
My school has lots of extracurricular activities				
Agree	2571	4625	Ref	Ref
Disagree	1325	2238	**1.56 [1.42–1.72]**	**0.84 [0.73–0.97]**
Do you know who provides mental health support in your school/college?				
Yes	1160	4025	Ref	Ref
No	1195	4379	**0.89 [0.81–0.97]**	**0.69 [0.61–0.79]**
I feel like I am part of my school				
Agree	718	4702	Ref	Ref
Disagree	1635	3704	**2.67 [2.43–2.93]**	0.99 [0.86–1.15]
My school deals well with racism				
Agree	710	4123	Ref	Ref
Disagree	1645	4281	**2.13 [1.94–2.34]**	**1.20 [1.04–1.38]**
I feel unfairly picked on by a teacher				
Rarely	858	4624	Ref	Ref
Often	1509	3768	**1.99 [1.82–2.17]**	**1.30 [1.14–1.47]**
Adults in my school listen when I share my opinion				
Often	1734	7476	Ref	Ref
Rarely	613	936	**2.68 [2.39–3.00]**	**1.21 [1.03–1.43]**
I worry about going to school/college				
Disagree	530	4998	Ref	Ref
Agree	1823	3408	**4.73 [4.29–5.22]**	1.11 [0.96–1.29]

*Note*: Schools *N* = 67, students *N* = 10,759. Variance of random effect for each school (Intercept) is 0.037, SD = 0.193. The univariable model uses the maximum available sample for each variable, while the multivariable model is restricted to participants with complete data across all included covariates (Aseltine & DeMartino, 2004; Duncan et al., 2019; Evans et al., 2019; Glennon et al., 2020; Groschwitz et al., 2017; Pierret et al., 2020; Schilling et al., 2014; Townsend et al., 2018; Wasserman et al., 2010). Values in bold are statistically significant *p* > 0.05.

Abbreviations: RCADS, Revised Anxiety and Depression Scale; (S)WEMWBS, the Short Warwick‐Edinburgh Mental Wellbeing Scale.

In the fully adjusted model, individual characteristics associated with a higher likelihood of SH included: gender as female (adj. OR = 1.15; 95% CI 0.99–1.32, *p* = 0.05), other (adj. OR = 3.49; 95% CI 2.38–5.12, *p* < 0.001) and prefer not to say (adj. OR = 2.02; 95% CI 1.44–2.83, *p* < 0.001) in comparison to males. Lower wellbeing scores (adj. OR = 0.93; 95% CI 0.93–0.95, *p* < 0.001), and higher RCADS scores (adj. OR = 1.12; 95% CI 1.11–1.13, *p* < 0.001) were associated with a higher likelihood of SH.

The total sample size (*N* = 12,554) represents participants in years 7–11 in the OxWell dataset. Of this total sample, 10,759 participants (85.7%) had complete data on the outcome variable, with 14.30% of the data missing across all participants. To account for missing data, we have defined the analytic sample as the subset of participants who have complete data not only for the outcome variable but also for all other covariates included in the regression analysis (Table 4). This resulted in a reduced sample size of 10,759 participants (85.7% of the total sample).

The percentage of missing data varied across individual variables, ranging from 10.1% missing data for Gender diverse (other and prefer not to say) to 14.30% for both School Year and RCADS. To determine whether missing data for specific variables was significantly associated with other characteristics in the sample, We used Chi Square tests for categorical variables, and *t*‐tests for continuous variables. The missingness of data for school year, RCADS, enjoyment, bullying, and worry were significantly associated with the SH outcome (*p* < 0.00).

## DISCUSSION

Our findings emphasise how important school is as a setting for identification of SH, given the substantial variation in levels of SH reported by students. However, our findings suggest that school characteristics are less important than individual student experience in relation to SH among pupils. We also found the expected and often reported associations with poorer mental health and gender, including gender diverse groups, who have been less studied.

The direction of the relationships between school experience and SH cannot be determined from our cross‐sectional study, given that data on both were collected at the same time. It may be that experiencing difficulties at school leads the student to SH as a coping strategy, or that an individual who self‐harms increasingly finds themself disconnected from school life, either due to stigma surrounding the behaviour, or because of the underlying factors that precipitated the initiation of self‐harming behaviours. Prospectively collected data are needed to replicate such findings longitudinally, this is not possible with the OxWell data, which are anonymised so individual pupil trajectories cannot be measured although some schools and therefore pupils have contributed to more than one survey. However, even small effects of school‐level influence on SH in young people are of public‐health importance, given the high prevalence of SH and the strong link with suicide as well as the fact that the vast majority of young people in the population are enroled in school.

A study on the influence of collated school level environment on student mental health in the MYRIAD trial (Ford et al., 2021) showed a small but important impact of schools on mental health related to the characteristics of the school and the school catchment area, including school climate which is potentially tractable. In the current study, several factors influencing the relationship between school experience and SH could potentially be addressed by schools, and indeed are important factors for schools to improve in their own right. To enhance the student perception of the school environment, measures can be taken to improve how incidents concerning bullying and racism are addressed, not only for the individual experiencing the bullying or racism, but for all students at the school as many are likely to be impacted by observing or hearing about such events (Clarkson et al., 2022). This emphasises the importance of whole‐school interventions, which are particularly effective in reducing bullying behaviour (Clarke et al., 2021).

There is also a longitudinal relationship between poor student‐teacher relationships and bullying and SH (Arseneault, 2018), so support for teachers to improve relationships, particularly with vulnerable students may benefit the student's mental health and engagement with school. Similarly, students perceiving teachers as fair has been shown as protective against SH (Carter et al., 2007).

Thus, a focus on improving teacher‐student interactions for students who perceive themselves as unfairly picked on by their teachers is therefore important in reducing SH. Additionally, fostering extracurricular activities might play an important role in creating a more inclusive and supportive atmosphere, particularly for marginalised communities including females and gender diverse young people. Extracurricular clubs and activities are an important part of many students' school experience, and at a systemic level, schools offering diverse extracurricular activities may enhance the school experience for students by prioritising both student wellbeing and academic success (Soneson et al., 2023). Finally, it is imperative for schools to explore strategies to support students who may be hesitant to disclose their gender or identify as part of a gender minority, as we found that these students showed markedly increased likelihood of engaging in SH.

### Methodological issues

Our study has several strengths, including a large sample of schools across England and the use of validated measures where these were available. The implementation of a multilevel model allowed for the analysis of how student SH behaviours were influenced by both individual characteristics, such as gender, and the school experience factors. By considering the school as a random intercept, the model acknowledged that students within the same school may have had more similar outcomes compared to students from different schools, which seems likely given the widely diverging levels of SH reported by students at different schools. Running multilevel models allowed us to understand whether differences in levels of reported SH were due to individual characteristics or because of differences between schools. Another strength of our research is the inclusion of gender diverse groups in the analyses, those who specified their gender as ‘other’ and ‘prefer not to say’, especially as these groups have shown higher proportions of SH compared to males and females but are under‐researched.

Inevitably there were also some limitations. Our more conservative approach to categorising the individually reported school experience variables reported in Table 1 aimed to ensure that we did not overestimate school experiences, whether positive or negative. In future analyses a larger sample would allow for ‘neither agree or disagree’ to be included in a logistic regression as a third level in the factor. This was not possible in the current study and led to convergence issues of the model due to the sample size. If a substantial proportion of students fall into the ‘Neither Agree nor Disagree’ category and if they have a higher probability of SH, grouping them with the ‘Disagree’ or ‘Agree’ category might underestimate the odds of SH for those individuals. Conversely, if they have a lower probability of SH, it might lead to an overestimation.

Although we have used an acceptable measure based on previous research (Hawton et al., 2002), there is a potential for over‐identification of SH due to the single‐item approach. Additionally, we excluded responses for those who said ‘prefer not to say’, which will have influenced the SH estimates. Furthermore, we recognise that excluding respondents who selected ‘prefer not to say’ could impact our findings, as this group may include students who SH but are reluctant to disclose this information. Students who selected the ‘prefer not to say’ option regarding their SH behaviour chose not to skip the question but intentionally withheld information. It is important to understand their reasons for this choice as SH is a sensitive topic area and students may not want to share information for various reasons. This could be explored through qualitative interviews and focus groups, or by offering students more privacy when completing questionnaires, rather than participating with other students in a classroom as this was often the case in the OxWell study.

Further education colleges were excluded from these analyses, but future research should repeat and extend the current analysis to FECs as these institutions cater for the emerging adult age‐group who have the highest prevalence of anxiety, depression and SH (McManus et al., 2019). Additionally, many school pupils may have SH behaviours that continue into college.

## CONCLUSION

In summary, this study has demonstrated that the levels of SH reported varies substantially between schools, and negative school experiences are associated with greater SH. Longitudinal studies of school experience and SH are required to provide stronger evidence that school experiences lead to SH. In the mean‐time Interventions that address bullying, racism, teacher‐pupil relationships as well as providing specific support for more vulnerable groups such as females and gender diverse young people are important components of public mental health interventions that might reduce levels of SH. If their implementation demonstrably reduced levels of SH in school, this would be powerful evidence of a causal link.

## AUTHOR CONTRIBUTIONS

**Rasanat Fatima Nawaz**: Conceptualization; data curation; formal analysis; investigation; methodology; project administration; resources; software; visualization; writing—original draft; writing—review and editing. **Tamsin Jane Ford**: Investigation; methodology; supervision; writing—review and editing. **Mina Fazel**: Conceptualization; data curation; funding acquisition; investigation; project administration; writing—review and editing. **Galit Geulayov**: Supervision; writing—review and editing. **Simon R. White**: Data curation; formal analysis; methodology; supervision; visualization; writing—review and editing.

## CONFLICT OF INTEREST STATEMENT

Tamsin Jane Ford's research group receives payments from Place2Be, a third sector organisation who deliver mental health training and interventions to schools across the UK, for research methodology consultation.

## ETHICAL CONSIDERATIONS

The OxWell Student Survey (2023) has been approved by the University of Oxford Research Ethics Committee (Reference: R62366/RE0014).

## Data Availability

The data used in this study are not publicly available. The study protocol and variable guides are available through the Open Science Framework (https://osf.io/sekhr/). Fully deidentified extracts of the data can be provided to academic research collaborators upon reasonable request after a review process by the research team to ensure that uses of the data fall under the remit of the intended purposes set out in the privacy information and to prevent duplication of analyses. The data are not publicly available because of ethical and information governance restrictions. The full list of questions as well as other details are available on a project‐specific OxWell Open Science Framework website (https://osf.io/sekhr/) along with the study protocol.
